# 血液肿瘤患者碳青霉烯类耐药肠杆菌科细菌（CRE）感染的诊治与防控中国专家共识（2025年版）

**DOI:** 10.3760/cma.j.cn121090-20250403-00162

**Published:** 2025-06

**Authors:** 中华医学会血液学分会 中国医师协会血液科医师分会 中华医学会细菌感染与耐药防治分会

## Abstract

细菌耐药已成为全球公共卫生领域的重大挑战，碳青霉烯类耐药肠杆菌科细菌（CRE）出现并在全球范围内快速播散，对人类健康构成极大威胁。血液肿瘤患者是CRE感染的高危人群，且感染的病死率亦较高。在血液肿瘤患者中，如何诊治和防控CRE感染已成为当前细菌感染领域最为棘手的问题。为规范我国血液肿瘤患者CRE感染诊治方案和防控策略，国内血液、感染等领域专家对CRE感染的流行病学特点、实验室检测、治疗原则、主要治疗药物和方案、CRE感染及定植的危险因素、医院感染防控等方面进行讨论，形成了本共识。

## Abstract (en)

Antimicrobial resistance in bacteria has become a major challenge in global public health. Globally, the emergence and rapid spread of carbapenem-resistant Enterobacteriaceae（CRE）pose a significant threat to human health. Patients with hematological malignancies are at a high risk of CRE infection, and the mortality rate of such infections is also relatively high. In these patients, how to diagnose, treat, and prevent CRE infection has become the most difficult issue in bacterial infection. To standardize the diagnosis, treatment plans, and prevention strategies for CRE infection in patients with hematological malignancies in China, hematology and infectious disease experts were invited to discuss and reach a consensus on the epidemiological characteristics of CRE infection, laboratory detection, treatment principles, main therapeutic drugs and dosing regimens, risk factors for CRE infection and colonization, and hospital infection prevention and control.

碳青霉烯类药物是治疗细菌感染，特别是肠杆菌科细菌感染的最强效的β-内酰胺类药物[1]。临床菌株一旦对碳青霉烯类药物产生耐药，治疗将面临极大困难。近年来，碳青霉烯类耐药的肠杆菌科细菌（CRE）感染的检出率逐渐升高。相较于碳青霉烯类药物敏感的肠杆菌科细菌（CSE），由于CRE感染的治疗方法有限，有效的抗感染治疗往往被延迟[2]，CRE感染患者的死亡率较高[2]–[3]。由于CRE感染的高危害性，2013年美国疾病控制与预防中心（CDC）将CRE列为耐药细菌“紧急”级别的首位，2024年世界卫生组织（WHO）同样将CRE列为耐药风险最高级别第一位。

血液肿瘤患者是发生CRE感染的高危人群[4]。这些患者由于原发性免疫缺陷和接受化疗、放疗、造血干细胞移植（HSCT）等治疗措施导致中性粒细胞缺乏等免疫功能缺陷，其CRE感染的检出率和病死率较其他科室患者更高[5]–[6]。制订针对血液肿瘤患者CRE感染的诊治与防控专家共识是血液科医师在临床工作中的迫切需要。2020年，由国内血液、感染等领域专家共同讨论制订的《血液肿瘤患者CRE感染的诊治与防控中国专家共识（2020年版）》发表，旨在为血液肿瘤患者CRE感染的诊治和防控提供参考意见。但近年来，CRE感染的流行病学特点、碳青霉烯酶基因型分布特点、防控措施及治疗药物均发生了改变，相关数据已更新。因此，需要对2020版共识重新进行修订。

一、CRE概述

1. CRE的定义：满足以下任一条件的肠杆菌科细菌：①对任一碳青霉烯类药物耐药［亚胺培南、美罗培南、多利培南的最低抑菌浓度（MIC）≥4 mg/L，或厄他培南MIC≥2 mg/L］；②产碳青霉烯酶；③如果是对亚胺培南天然耐药的细菌（如摩氏摩根菌、变形杆菌属、普罗威登斯菌属），必须对其他碳青霉烯类药物（如美罗培南、厄他培南、多利培南）耐药[7]。

2. CRE的发生机制[7]：①产生碳青霉烯酶，水解碳青霉烯类抗菌药物；②外膜孔道蛋白缺失或表达降低，导致抗菌药物不能透过细胞膜进入细菌内，通常合并AmpC头孢菌素酶或超广谱β-内酰胺酶（ESBL）的生成过多；③编码外排泵的基因过度表达，导致抗菌药物清除增加；④青霉素结合蛋白的结构改变，导致其与碳青霉烯类药物的亲和力下降。其中，①和②是主要机制。

依据Ambler分类系统将β-内酰胺酶分为4类：A、B、C、D。其中A、B、D类为碳青霉烯酶[7]–[8]（表1），而C类为头孢菌素酶。

**表1:** 碳青霉烯酶的分类[7]–[8]

分类	活性位点	代表酶	常见细菌
A类	丝氨酸	KPC、SME、GES型	肠杆菌科细菌，铜绿假单胞菌少见
B类	锌	NDM、VIM、IMP型	肠杆菌科细菌，铜绿假单胞菌，不动杆菌属少见
D类	丝氨酸	OXA-48、OXA-23、OXA-24型	部分肠杆菌科细菌（以OXA-48为主），不动杆菌属（OXA-23、OXA-24）

二、CRE感染的流行病学特征

1. CRE感染的检出率：自2000年以来，CRE感染的检出率逐年增加，但不同国家及地区CRE感染的检出率存在差异。2001年美国CRE感染的检出率为1.2％，2011年则升至4.2％[9]。2010–2013年欧洲CRE感染的检出率为2.0％[10]。2014–2017年欧洲碳青霉烯类耐药大肠埃希菌（CREC）感染的检出率为0.1％～0.2％，碳青霉烯类耐药肺炎克雷伯菌（CRKP）感染的检出率为6.8％～7.4％，其中希腊最高，达64.7％，其次为意大利（29.7％）和波兰（22.5％）[11]。与欧美国家相比，我国CRE感染的检出率更高，2014年为12.5％，2016年为22.9％[12]，2019年则升至26.8％，2024年为23.4％（CHINET监测网资料）。且各省市CRE感染检出率差异很大，河南最高（199/935），其次为上海（184/935）、重庆（105/935），青海和西藏最低（0/935）。在分离菌株中，最常见的为肺炎克雷伯菌，其次为大肠埃希菌[13]–[14]。

血液肿瘤患者和接受HSCT的患者是CRE感染的高危人群，且其检出率逐年增加。在血液肿瘤合并肠杆菌科细菌所致血流感染（BSI）的患者中，欧美CRE感染的检出率为4.7％～23.1％[3],[15]，我国不同地区、不同疾病血液肿瘤患者CRE感染的检出率不同（表2）。在HSCT后发生肠杆菌科细菌所致BSI的患者中，欧洲CRE感染的检出率为8.4％～10.0％[2],[23]，我国为6.2％～10.4％[24]–[26]。异基因造血干细胞移植（allo-HSCT）（15.8％～23.7％）高于自体造血干细胞移植（auto-HSCT）（4.8％～8.9％）[2],[23]，替代供者移植高于同胞相合移植[24]–[26]。

**表2:** 我国血液恶性肿瘤患者CRE感染检出率[16]–[22]

研究单位	时间	人群	部位	患者例数	CRE感染检出率
安徽省立医院[16]	2010-2014年	血液病（恶性85.6％，非恶性14.4％）	–	158	CREC：0.8％；CRKP：11.8％
福建医科大学附属协和医院[17]	2013-2016年	血液恶性肿瘤化疗后	BSI	250	CREC：2.7％；CRKP：12.2％
中国医学科学院血液病医院[18]	2014-2018年	血液病 ①恶性87.2％，非恶性10.8％ ②粒细胞缺乏87.9％ ③化疗78.3％，HSCT 11.4％	BSI	1098	CREC：3.9％；CRKP：5.7％
南方医科大学南方医院血液科[19]	2018-2021年	恶性血液病（化疗70.6％，HSCT 25.8％）	BSI	328	CREC：7.9％；CRKP：10.4％
解放军总医院第五医学中心[20]	2019-2021年	血液病（恶性94.1％，非恶性5.9％）	BSI	221	CREC：15.5％；CRKP：21.5％
广东50家医院血液科[21]	2019年	血液病	BSI	725	CRKP：儿童3.7％，成人14.1％；CREC：儿童4％，成人7.9％
广东56家医院血液科[22]	2020-2024年	血液病	BSI	4353 （2020年837，2021年814，2022年874，2023年917，2024年911）	CRKP：2020-2024年从5.8％上升至15.8％；CREC：2020-2024年从6.7％上升至14.0％

**注** CRE：碳青霉烯类耐药的肠杆菌科细菌；HSCT：造血干细胞移植；BSI：血流感染；CREC：碳青霉烯类耐药的大肠埃希菌；CRKP：碳青霉烯类耐药的肺炎克雷伯菌；–：无数据

欧美国家血液肿瘤患者CRE感染的菌株类型以肺炎克雷伯菌（70.0％～80.0％）为主，其次为大肠埃希菌（5.0％～20.0％）[2],[23]。但我国血液肿瘤患者CRE感染的菌株类型中大肠埃希菌（56.0％～58.1％）和肺炎克雷伯菌（36.0％～41.9％）几乎各占一半[26]–[28]。在成人患者中，近期全国92家医院的筛查结果也显示，CRE菌株类型中大肠埃希菌（44.4％）和肺炎克雷伯菌（41.3％）几乎各占一半[29]。在儿童患者中，中国医学科学院血液病医院的结果显示，CRE感染菌株主要为大肠埃希菌（53.8％），其次为阴沟肠杆菌（24.0％）和肺炎克雷伯菌（22.5％）[30]。

2. CRE的分子流行病学特征：我国CRE以产KPC酶及NDM酶为主，部分产IMP酶和OXA酶。不同CRE菌株的产酶方式不同（表3）。在血液病患者中，我国CRE以产NDM酶（73.0％）及KPC酶（21.9％）为主，部分产IMP酶（2.1％）和OXA酶（1.4％）。大肠埃希菌以产NDM酶（98.6％）为主，肺炎克雷伯菌以产KPC酶（67.5％）为主，其次为NDM酶（27.0％）[29]。KPC酶与NDM酶均属于碳青霉烯酶，均可以水解大多数β-内酰胺药物，包括碳青霉烯类。不同之处在于，KPC酶为丝氨酸酶，可以水解氨曲南，但能被新型酶抑制剂阿维巴坦、瑞来巴坦和法硼巴坦抑制；而NDM酶为金属酶，不能水解氨曲南，但不被阿维巴坦、瑞来巴坦和法硼巴坦等抑制[10]。近年来逐渐出现关于bla_KPC_基因突变体的报道，与野生型bla_KPC_相比，氨基酸序列的替代、插入或删除改变了KPC的结构，增强了其对头孢他啶的亲和力，减弱了其对阿维巴坦的亲和力，从而介导细菌对头孢他啶/阿维巴坦（CZA）的耐药性。截至2023年底，全世界已报道发现150种bla_KPC_基因突变体。在中国，新的突变体主要来自bla_KPC-2_；而在美国和欧洲，新的突变体主要来自bla_KPC-2_和bla_KPC-3_[32]。

**表3:** 我国CRE的分子流行病学特征[13],[31]

人群	所有CRE	大肠埃希菌	肺炎克雷伯菌	阴沟肠杆菌
总体	产KPC酶54.3％～62.2％； 产NDM酶32.1％～35.7％； 产OXA-48酶7.3％	产NDM酶93.8％～96.0％	产KPC酶64.5％～84.9％； 产NDM酶12.5％～21.1％； 产OXA-48酶0.2％～9.6％	产NDM酶72.9％～75.0％； 产IMP酶13.9％～16.3％； 产KPC酶4.7％～8.3％
成人	产KPC酶70.3％； 产NDM酶20.6％	产NDM酶93.0％	产KPC酶87.0％； 产NDM酶5.4％	–
儿童	产KPC酶35.1％； 产NDM酶49.0％； 产OXA-48酶13.3％	产NDM酶97.2％	产KPC酶44.7％； 产NDM酶34.9％； 产OXA-48酶17.5％	–

**注** CRE：碳青霉烯类耐药的肠杆菌科细菌；–：无数据

三、CRE感染的危险因素和临床预后

血液肿瘤患者发生CRE感染的危险因素包括：CRE定植，既往CRE感染，既往使用碳青霉烯类、酶抑制剂复合制剂、喹诺酮类、氨基糖苷类和头孢菌素类抗菌药物，老年患者，入住ICU，侵袭性操作，长期住院，长时间（≥7 d）中性粒细胞缺乏和接受allo-HSCT等[2]–[3],[33]–[35]。此外，长期中性粒细胞缺乏、出现消化道症状、肛周感染、黏膜炎、接受放化疗和多部位CRE定植是CRE定植者发生BSI的危险因素[26],[28],[30],[36]–[37]。

由于血液肿瘤患者和接受HSCT的患者存在中性粒细胞缺乏、免疫抑制剂应用等导致的免疫功能缺陷，因此CRE感染导致的病死率高[28],[38]。文献报道，在血液肿瘤合并BSI的患者中，CRE所致30 d相关病死率高达50％～76.5％[3],[27],[34],[38]–[39]；在接受HSCT患者中，CRE感染患者3个月的总体病死率为58％～73.8％，allo-HSCT明显高于auto-HSCT[40]–[41]。

四、CRE的表型和基因型检测[42]

CRE所产碳青霉烯酶的表型检测对于CRE感染精准治疗至关重要。随着检测技术提高，可实现“先酶后敏”。常见酶型和基因型检测方法如下：

1. 表型初筛实验：主要有以下2种：①纸片扩散法：美罗培南（每片10 µg）或亚胺培南（每片10 µg）纸片抑菌圈直径≤22 mm；②肉汤稀释法或E-test法：美罗培南或亚胺培南MIC≥2 mg/L。

2. 表型确证实验：主要有以下3种：①改良碳青霉烯灭活试验（mCIM）；②Carba NP试验；③EDTA-碳青霉烯灭活试验（eCIM）。其中mCIM和eCIM是国内最常用的检测方法。mCIM和Carba NP试验主要用于检测产碳青霉烯酶，而eCIM主要用于检测产金属酶。

3. PCR或mNGS检测：主要检测KPC、NDM、OXA-48、IMP和VIM等五种重要的碳青霉烯酶基因。

建议有条件的医疗机构对CRE分离株开展碳青霉烯酶表型或基因型的检测，尤其在不能获得CZA等新型β-内酰胺酶抑制剂复合制剂等抗菌药物的药敏试验结果时[43]。对于部分无法进行酶型表型检测的医疗机构，可基于药敏结果和上述耐药机制，予以较恰当的药物选择。

五、CRE感染的抗菌药物治疗

CRE感染的治疗原则：①临床标本中分离到或分子学手段检测到CRE，当痰标本中检出CRE时，首先应结合临床表现区分是感染还是定植，然后再决定是否启动抗菌药物治疗。②尽量根据药敏结果选择敏感抗菌药；或选择中介或接近中介或有一定抑菌圈的抗菌药，一般足剂量联合治疗；有条件的实验室应检测联合药敏或CRE的分子耐药表型，实现精准治疗。③根据PK/PD原理设计给药方案，如增加给药剂量、延长抗菌药的滴注时间。④肝肾功能异常、老年患者，抗菌药物的剂量应作适当减量。⑤消除感染危险因素，积极处理原发疾病。⑥抗菌药物治疗的疗程取决于感染的严重程度、基础疾病、抗菌药物对CRE菌株的杀菌作用等多方面因素[44]。

（一）治疗CRE感染的常用抗菌药物

治疗CRE感染的常用抗菌药物见表4[44]–[48]。

**表4:** 治疗CRE感染的常用抗菌药物的推荐剂量及注意事项[44]–[48]

抗菌药物	类别及作用机制	推荐成人剂量	推荐儿童剂量	不同部位CRE感染的用药推荐	常见不良反应	注意事项
头孢他啶/阿维巴坦（CZA）	新型β-内酰胺酶抑制剂	①成人：2.5 g（2 g/0.5 g）每8 h 1次，每次输注3 h； ②与氨曲南联合应用：CZA 2.5 g，每8 h 1次，输注>3 h；氨曲南2.0 g，每8 h 1次，输注>3 h，与CZA同时输注（建议使用Y型管）	①3～6个月：头孢他啶40 mg/kg+阿维巴坦10 mg/kg，每8 h 1次，输注>2 h； ②6个月～2岁：头孢他啶50 mg/kg+阿维巴坦12.5 mg/kg，每8 h 1次，输注>2 h； ③2～18岁：头孢他啶50 mg/kg（最大2.0 g）+阿维巴坦10 mg/kg（最大0.5 g）每8 h 1次，输注>2 h	①复杂性腹腔感染（cIAI）； ②医院获得性肺炎（HAP）； ③机械通气相关性肺炎（VAP）； ④在治疗方案选择有限的成人患者中治疗由下列对本品敏感的革兰阴性菌引起的感染：肺炎克雷伯菌、阴沟肠杆菌、大肠埃希菌、奇异变形杆菌和铜绿假单胞菌	①过敏反应； ②中枢神经系统反应，如癫痫发作	①对于产KPC酶和OXA-48酶的CRE敏感，是治疗这类CRE感染的首选药物； ②对于产NDM酶、VIM酶和IMP酶的CRE无效，对于此类感染可以考虑联合应用氨曲南； ③在肺泡上皮衬液中的浓度较高； ④有较好的血脑屏障穿透率，常规剂量静脉给药即可达到有效组织体液浓度及较好的临床疗效
多黏菌素E、多黏菌素B	阳离子多肽类抗生素； 作用机制：破坏细胞膜完整性，使细胞内的主要成分外流，最终使细胞裂解死亡	①甲磺酸多黏菌素E：负荷量9 MU，随后9 MU/d，分2～3次给药。 ②硫酸多黏菌素E：100万～150万U/d，分2～3次给药； ③多黏菌素B：负荷量2.5 mg/kg，随后1.5 mg/kg，每12 h 1次	①甲磺酸多黏菌素E：负荷剂量5 mg/kg（最大300 mg CBA），维持剂量2.5 mg/kg，每12 h 1次（最大180 mg CBA）； ②多黏菌素B：负荷剂量2.5 mg/kg，维持剂量1.5 mg/kg，每12 h 1次（最大200 mg/d）	①血流感染（BSI）； ②脑膜炎； ③肺部感染； ④泌尿系统感染（UTI）； ⑤皮肤软组织感染（SSTI）	①肾毒性； ②神经系统毒性	①异质性耐药较常见； ②肺组织浓度较低，治疗肺炎需辅以雾化吸入。硫酸多黏菌素E雾化吸入：50～75 mg/次，溶于3～4 ml生理盐水中，每日2次雾化吸入。尽量用震动筛孔雾化器进行雾化； ③脑脊液浓度低，可以多黏菌素E鞘内或脑室内给药：3.2～10 mg/d，不能超过20 mg/d； ④甲磺酸多黏菌素E可以从肾脏清除，并在膀胱内转化为多黏菌素E，因此尿液浓度高，可以用于治疗UTI； ⑤多黏菌素B尿液浓度低，不用于治疗UTI； ⑥肾功能损害时：多黏菌素B不需要调整剂量，甲磺酸多黏菌素E需要调整剂量；老年人及肾功能损害者需要监测肾功能和尿常规
氨曲南/阿维巴坦	新型β-内酰胺酶抑制剂	首次负荷剂量2.67 g（2 g / 0.67 g），输注3 h；之后维持剂量2 g（1.5 g / 0.5 g）每6 h 1次，每次输注3 h	–	用于治疗成人患者以下感染： ①cIAI； ②HAP包括VAP； ③复杂尿路感染（cUTI），包括肾盂肾炎； ④治疗方案选择有限的成人革兰阴性菌引起的感染	①贫血； ②腹泻； ③丙氨酸转氨酶（ALT）升高，天冬氨酸转氨酶（AST）升高； ④大多数不良反应为轻中度	①对于产金属酶的CRE感染者，可以首选氨曲南/阿维巴坦； ②对KPC突变菌株仍有较高的体外敏感性； ③轻度肾功能损害（50 ml/min<预估CrCl≤80 ml/min）无需调整剂量，中重度肾功能损害需减量应用； ④肝功能损害患者无需调整剂量
依拉环素	氟环素类抗生素，属于四环素类； 作用机制：可通过与细菌核糖体30S亚单位结合，阻止氨基酸残基整合，从而干扰细菌蛋白质的合成，为抑菌剂	1 mg/kg每12 h 1次，输注>60 min	–	①成人cIAI	①肝功能异常； ②淀粉酶升高； ③凝血功能异常	①对铜绿假单胞菌无抗菌活性； ②在肺泡上皮衬液和肺泡巨噬细胞内浓度高，肺组织浓度高； ③肾功能不全无需调整剂量； ④对轻度至中度肝损伤患者无需调整剂量；对重度肝损伤（Child Pugh C级）患者，需要减量（第1天：1 mg/kg每12 h 1次，第2天：1 mg/kg每日1次）
替加环素	甘氨酰环素类抗生素； 作用机制：通过与细菌核糖体30S亚单位结合，从而抑制细菌蛋白质合成，为抑菌剂	①标准剂量：负荷量100 mg，随后50 mg每12 h 1次； ②高剂量：负荷量200 mg，随后100 mg每12 h 1次	≥8岁：负荷剂量4 mg/kg（最大200 mg），维持剂量2～3.2 mg/kg，每12 h 1次（最大100 mg）	①cIAI； ②社区获得性肺炎（CAP）； ③SSTI	①肝功能异常； ②淀粉酶升高； ③胃肠道反应	①对铜绿假单胞菌无抗菌活性； ②需要联合应用，如：多黏菌素、磷霉素、氨基糖苷类、碳青霉烯； ③在肺泡细胞、上皮细胞衬液、炎性渗出液、胆囊和结肠中有较高的浓度，但血浆和尿液中浓度低。 a. 治疗UTI：不推荐使用；b. 治疗BSI：不推荐使用；c. 治疗HAP和VAP：需要高剂量，并与其他药物联合应用； ④肾功能不全无需调整剂量； ⑤轻度肝功能损害无需调整剂量，重度肝功能损害需要调整剂量（负荷量100 mg，随后25 mg每12 h 1次）
亚胺西瑞（亚胺培南/西司他丁/瑞来巴坦）	新型β-内酰胺酶抑制剂	1.25 g每6 h 1次，输注>30 min	①2～12岁：15 mg/kg亚胺培南（最大0.5 g）+7.5 mg/kg瑞来巴坦（最大0.25 g）每6 h 1次，输注>30 min； ②12～18岁：0.5 g亚胺培南+0.25 g 瑞来巴坦，每6 h 1次，输注>30 min	①cUTI； ②cIAI； ③HAP/VAP	–	仅对产KPC酶的CRE有效
美罗培南/法硼巴坦	新型β-内酰胺酶抑制剂	4 g每8 h 1次，输注>3 h	40 mg/kg美罗培南+40 mg/kg法硼巴坦（最大2.0 g）每8 h 1次，输注>3 h	①cUTI； ②cIAI； ③HAP/VAP； ④与上述任何感染相关或疑似相关的菌血症； ⑤治疗方案选择有限的成人患者中革兰阴性菌引起的感染（EDA）	–	仅对产KPC酶的CRE有效
磷霉素	磷酸烯醇丙酮酸类似物； 作用机制：抑制肽聚糖合成的磷酸烯醇丙酮酸转移酶（MurA）的活性，影响细菌细胞壁早期合成，属于繁殖期快速杀菌剂	6～8 g每8 h 1次	–	①非复杂尿路感染； ②呼吸道感染； ③SSTI； ④与其他药物联合治疗重度感染，如BSI、腹膜炎、骨髓炎； ⑤对于CRE感染，仅用于联合方案，可以作为任何感染部位的第三种药物选择	①胃肠道反应	①对鲍曼不动杆菌无抗菌活性； ②广泛分布于组织、体液中，尿液中浓度最高，并可有效穿透至脑脊液
亚胺培南、美罗培南	碳青霉烯类	①亚胺培南MIC为≤8 mg/L时： 1 g每6～8 h 1次，且需延长输注时间至2～2.5 h； ②美罗培南MIC为≤8mg/L时： 2 g每8 h 1次，且需延长输注时间至2～4 h或持续输注	①亚胺培南MIC为≤8 mg/L时： a. 2～12岁：15 mg/kg，每6 h 1次（最大0.5 g）； b. 12～18岁：0.5 g每6 h 1次，输注>30 min ②美罗培南MIC为≤8 mg/L时：40 mg/kg，每8 h 1次，输注>3 h（最大2.0 g）	①亚胺培南：腹腔感染、肺炎、BSI、泌尿生殖系统感染、SSTI、骨关节感染、心内膜炎、妇科感染； ②美罗培南：腹腔感染、肺炎、UTI、妇科感染、BSI、SSTI及中枢神经系统感染	①过敏反应； ②胃肠道反应； ③中枢神经系统反应，如癫痫发作	①MIC≤8 mg/L时，与另一种体外有抗CRE活性的药物联合应用；碳青霉烯类药物需要大剂量给药，并延长静脉滴注时间至2～4 h； ②MIC>8 mg/L时，碳青霉烯类药物无效

**注** CRE：碳青霉烯类耐药的肠杆菌科细菌；CBA：黏菌素活性基质；CrCl：肌酐清除率；MIC：最低抑菌浓度；–：无数据

（二）CRE感染的抗菌治疗策略

1. CRE感染的经验性治疗[49]–[50]：在启动经验性治疗前需要进行风险评估[37],[51]–[54]（表5）。

**表5:** CRE经验性治疗前的风险评估[37],[51]–[54]

高风险：同时具备1和2的任意一项
1. CRE主动筛查阳性患者：具备任意一项CRE感染的危险因素
①重度中性粒细胞缺乏（ANC<0.1×10^9^/L）预计持续≥7 d ②胃肠道黏膜炎 ③肛周感染 ④ICU入住 ⑤除肠道定植外，其他部位存在CRE定植
2. 严重的临床合并症：具备任意一项 ①休克或严重的脓毒症 ②呼吸衰竭：脱氧PaO_2_<60 mmHg或需要机械通气 ③弥散性血管内凝血 ④意识障碍或精神异常 ⑤需要治疗的充血性心力衰竭 ⑥需要治疗的心律失常 ⑦肾功能衰竭：肌酐清除率<30 ml/min或需要透析

**注** CRE：碳青霉烯类耐药的肠杆菌科细菌

（1）适用人群：同时满足下述3个条件的患者，可以考虑进行经验性治疗。

①CRE主动筛查阳性的患者或既往CRE感染的患者或局部有CRE流行（近期住院患者中CRE检出率>20％）；

②出现发热或其他可能的感染症状和体征；

③风险评估为高风险（表4）。

（2）初始经验性抗CRE治疗方案：

①需要覆盖假单胞菌和其他常见的革兰阴性菌［如产超广谱β-内酰胺酶（EBSL）的肠杆菌科细菌］。

②需要覆盖CRE：可以根据CRE主动筛查或既往CRE感染时的微生物学检测结果，并根据CRE的局部流行情况进行选择，可参考表4。

（3）初始经验性抗CRE治疗的再次评估（图1）[55]：

**图1:**
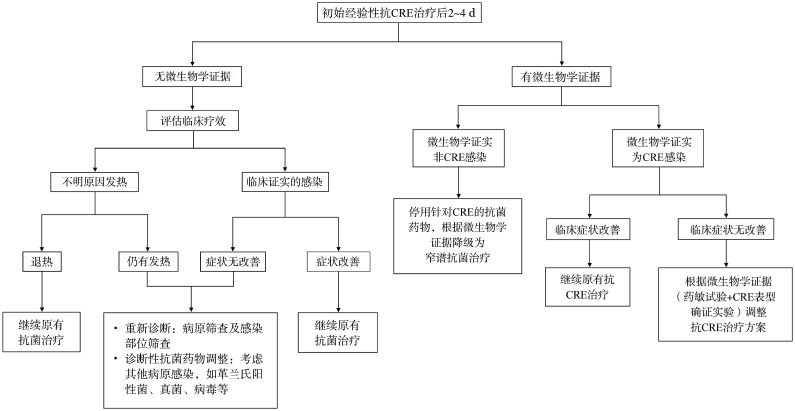
初始经验性抗碳青霉烯类耐药的肠杆菌科细菌（CRE）治疗的再次评估

①评估时间：初始经验性抗CRE治疗2～4 d后。

②评估指标：临床疗效和微生物学证据。

2. CRE感染的目标治疗（图2）[44]–[51]：

**图2:**
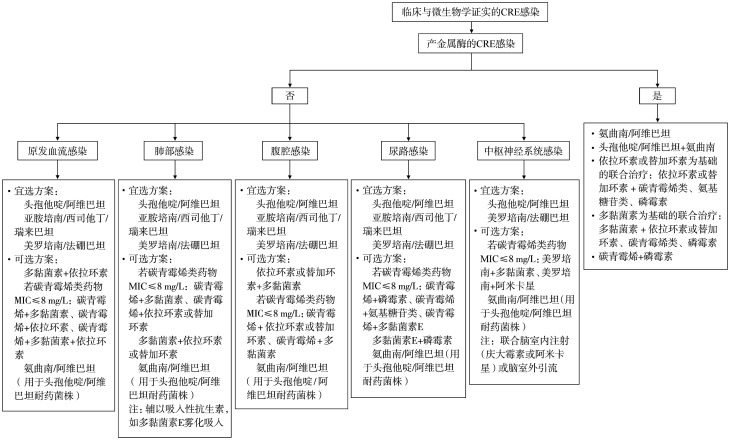
基于不同感染部位和不同碳青霉烯类耐药的肠杆菌科细菌（CRE）表型的目标治疗推荐 MIC：最低抑菌浓度

（1）适用人群：临床与微生物学证实的CRE感染患者，即有感染的临床症状和体征、感染部位分离到CRE。

（2）抗菌药物选择：依据微生物学检测结果选择具有体外活性的抗菌药物单药或联合应用（表6）。同时需要考虑CRE的表型和感染部位（图2）。

**表6:** 抗CRE感染的常用联合治疗方案[44]–[51]

治疗方案	注意事项
两药联合治疗方案： 头孢他啶/阿维巴坦+氨曲南 多黏菌素+依拉环素或替加环素 多黏菌素+磷霉素 依拉环素或替加环素+磷霉素 碳青霉烯类+多黏菌素 碳青霉烯类+依拉环素或替加环素	①头孢他啶/阿维巴坦+氨曲南主要用于治疗产金属酶的CRE感染，两药需同步输注，方可保持协同效应；有条件使用氨曲南/阿维巴坦^a^则优选单药。亚胺培南/西司他丁/瑞来巴坦+氨曲南在体外联合药敏试验中有协同作用，但无临床数据 ②如果碳青霉烯类药物MIC≤8 mg/L，可以选择碳青霉烯类药物与其他药物联合
三药联合治疗方案： 多黏菌素+依拉环素或替加环素+碳青霉烯类 多黏菌素+磷霉素+碳青霉烯类 多黏菌素+依拉环素或替加环素+磷霉素	①多黏菌素+依拉环素或替加环素+碳青霉烯类药物可用于CRE的严重感染如脑膜炎、心内膜炎、血流感染等

**注** CRE：碳青霉烯类耐药的肠杆菌科细菌；MIC：最低抑菌浓度；^a^氨曲南/阿维巴坦已在欧盟批准上市，2025年即将在中国上市

目前中国已上市治疗CRE感染的药物包括：多黏菌素B、甲磺酸多黏菌素E、替加环素、磷霉素、CZA和依拉环素。即将上市的药物有氨曲南/阿维巴坦、亚胺西瑞（亚胺培南/西司他丁/瑞来巴坦）和美罗培南/法硼巴坦。若碳青霉烯MIC≤8 mg/L，可以通过提高碳青霉烯类药物剂量、延长输注时间，并与其他有抗菌活性的药物联合应用达到抗CRE的目的[31],[44]–[46]。

①对产KPC酶或OXA-48酶的CRE感染患者：

a. CZA单药应用：对产KPC酶和产OXA-48酶的CRE感染有效。临床有效率优于其他抗菌药物的联合治疗方案（如以多黏菌素为基础的联合方案、碳青霉烯类药物为基础的联合方案等）[56]–[59]。

b. 亚胺西瑞单药应用：仅对产KPC酶的CRE感染有效。单药治疗有效率可达70％。其临床有效率和生存率均优于亚胺培南/西司他丁+多黏菌素联合方案[60]。

c. CZA与其他抗菌药物联合应用是否优于其单药应用存在争议。荟萃分析显示两组患者微生物清除率和死亡率均无明显差异[61]。

d. 除CZA和亚胺西瑞外，其他抗菌药物的联合治疗方案优于单药治疗方案。若碳青霉烯类药物的MIC≤8 mg/L，包含碳青霉烯类药物的联合方案优于不含碳青霉烯类药物的联合方案[62]–[64]。

②对产金属酶的CRE感染患者：依据药敏试验选择有抗菌活性的药物进行联合治疗。

a. CZA+氨曲南：体外研究和多项临床研究证实，CZA+氨曲南在体外有协同作用，可以有效抑制产金属酶CRE菌株的生长[65]–[66]；在体内CZA+氨曲南也可以有效治疗产金属酶CRE所致的BSI，降低死亡率，其疗效优于其他治疗方案[67]–[69]。

b. 氨曲南/阿维巴坦：针对金属酶治疗时，CZA+氨曲南治疗方案可能存在阿维巴坦剂量不足的问题。即将在中国上市的氨曲南/阿维巴坦是目前国内第一个可单药治疗产金属酶CRE的新型β-内酰胺酶抑制剂复合制剂。研究显示，氨曲南/阿维巴坦治疗产金属酶的革兰阴性菌感染者，临床治愈率和全因死亡率均优于对照组接受最佳可及方案（BAT）治疗的患者[70]。

c. 亚胺西瑞+氨曲南：亚胺西瑞+氨曲南在体外联合药敏试验中有协同作用，但无临床数据[71]。

d. 美罗培南/法硼巴坦+氨曲南：美罗培南/法硼巴坦+氨曲南在体外联合药敏试验中有协同作用，但无临床数据。

③本共识推荐：

a. 对产KPC酶或OXA-48酶的CRE感染患者：首选CZA单药治疗或亚胺西瑞（仅用于产KPC酶的CRE感染者）；若选择其他药物，则需要联合使用；碳青霉烯类药物MIC≤8 mg/L，可选择以碳青霉烯类药物为基础的联合治疗方案。

b. 对产金属酶的CRE感染患者：首选氨曲南/阿维巴坦，其次可选择CZA+氨曲南或其他抗菌药物联合治疗方案。

（3）抗菌药物应用过程中的耐药问题：CRE感染在CZA应用过程中出现耐药的问题正日趋显现。研究表明，接受非足量的CZA治疗是产生bla_KPC_突变体的主要危险因素。如KPC-33，此类突变体对CZA耐药，对碳青霉烯类药物敏感。另外还有部分变异体对CZA和碳青霉烯类药物均出现耐药。因此，对初始明确为CZA敏感的CRE感染者，应用CZA治疗过程中出现疗效由好变差时，需及时做细菌培养或检测耐药表型，经验性治疗可考虑CZA+碳青霉烯类药物、多黏菌素+依拉环素或替加环素或其他新型β-内酰胺类药物/β-内酰胺酶抑制剂复合制剂（如氨曲南/阿维巴坦、亚胺西瑞和美罗培南/法硼巴坦）[32],[72]。

六、CRE医院感染的预防和控制措施[73]–[74]

CRE感染的增多是抗菌药物选择压力、耐药基因水平传播和耐药克隆菌株传播共同作用的结果。必须将医院感染防控措施与抗菌药物临床应用管理相结合才能有效阻遏CRE的传播、减少耐药菌感染。

（一）CRE的监测

1. CRE的主动筛查（强推荐）[74]：

（1）定义：通过对无症状患者的标本进行培养、检测，发现CRE定植者。

（2）筛查人群：①存在CRE感染的危险因素；②患者从CRE流行区域转入；③患者入住医院或病区局部有CRE流行；④患者同一病房或同一病区其他患者为CRE定植或感染者[29],[35],[40],[49],[75]。

（3）筛查标本：粪便是最佳的筛查标本，如不易留取可以选择直肠拭子标本，其次是肛周拭子标本，样本的留取和送检参考2017年WHO指南[74]。

（4）筛查频率：推荐高危患者入院时及入院后每周进行一次CRE主动筛查，连续4周。研究显示，与单次筛查相比，连续多次筛查CRE定植检出率更高，接受医院感染的预防和控制措施后，CRE感染发生率降低，临床预后改善[76]。

2. 环境中CRE的筛查（常规推荐）：

（1）筛查范围：①CRE定植或感染者，及参与医疗工作的医护人员所接触的周围环境；②CRE高流行区域（近期住院患者中CRE检出率>20％）内的周围环境。

（2）检测方法：见CRE的主动筛查。

（二）对CRE定植或感染者实施的医院感染的预防和控制措施[73]–[74]

1. 手卫生（强推荐）：

（1）适用情况：在接触患者前后，实施清洁或无菌操作前后，接触患者血液体液后，接触患者周围环境后。

（2）方式：包括洗手和手消毒。同时强调戴手套不能替代手卫生，在戴手套前和脱手套后应执行手卫生。

2. 接触性预防和隔离（强推荐）：对CRE主动筛查阳性患者执行接触性预防和隔离措施可以降低院内CRE的传播[77]。

（1）隔离措施：①患者安置：将患者安置在单人房间；当条件受限时，可将感染或定植相同病原体的患者安置在同一病房；②设立隔离标识；③诊疗用品应专人专用；④医护人员对患者实施诊疗护理操作时应戴手套和穿隔离衣；⑤限制患者的转运。

（2）隔离期限：目前尚不明确。对于CRE定植者，原则上应隔离至至少连续2次主动筛查为阴性（间隔为每周1次）；对于CRE感染者，原则上应隔离至感染的症状好转或治愈，且CRE培养连续2次阴性。

3. 环境表面清洁（强推荐）：对CRE定植者或感染者、护理该患者的医护人员频繁接触的物体表面进行定期、充分清洁。通常以次氯酸盐作为环境清洁剂。环境表面清洁后，要留取环境中各部位的标本进行CRE筛查。出现CRE流行的区域，应暂时关闭病房，并进行彻底环境清洁。

4. 去定植措施：目前去定植措施作用尚不明确[73]–[74]。

（1）全身洗必泰擦浴：通常使用2％的洗必泰溶液每日全身擦拭。对CRE定植者，全身擦浴的去定植疗效尚不明确。

（2）肠道去定植：

①口服抗生素：通常予庆大霉素（每次80 mg，每日4次，口服）+多黏菌素E（每次1×10^6^，每日4次，口服）。目前口服抗生素进行CRE去定植仍存在争议，尤其是停药后复发，且还存在诱导细菌耐药的风险。因此作用尚不明确，需要更多的循证医学证据[78]–[79]。

②粪菌移植：小样本的研究显示，粪菌移植可以通过改变肠道微生态达到清除CRE定植的目的[80]–[81]。但粪菌移植目前还没有标准化的制备方法，且会增加感染风险。目前作用尚不明确。

（3）噬菌体去定植：一些动物研究、病例报告和小样本的临床研究显示，噬菌体用于肠道CRE去定植是有效和安全的[82]。但目前作用尚不明确。

（三）加强抗菌药物的管理和教育[73]

1. 严格掌握抗菌药物的应用指征和应用疗程（强推荐）：制定各级医疗机构的治疗指南或方案，严格掌握抗菌药物的应用指征和应用疗程，限制不必要的、长时间的、特定抗菌药物的应用。

2. 抗菌药物的轮换（作用尚不明确）：在防控CRE感染方面，抗菌药物轮换策略证据不足，仅慎重推荐在特定病区执行抗菌药物轮换策略。

七、结语

CRE感染的检出率呈逐年上升趋势，CRE感染已经成为公共卫生安全的重大威胁。血液肿瘤患者是CRE感染的高危人群，且CRE感染相关死亡率较高。本共识根据更新的研究结果，对血液肿瘤患者CRE感染的诊断和治疗策略进行了修改。同时对感染控制的相关措施提出建议，包括主动筛查、手卫生、接触性隔离、环境消毒和加强抗菌药物的管理等，以遏制CRE感染的发生和传播。
